# Supplementary material for: Translational Regulation of Pmt1 and Pmt2 by Bfr1 Affects Unfolded Protein O-Mannosylation
Source: Int J Mol Sci. 2019 Dec 10;20(24):6220. doi: 10.3390/ijms20246220 (PMC6940804; doi:10.3390/ijms20246220)
Supplement: Supplementary file 1 [file ijms-20-06220-s001.zip › ijms-656847-for XML-supplementary/ijms-656847-for proofreading-supplementary_NR.pdf]

Supplemental Figures

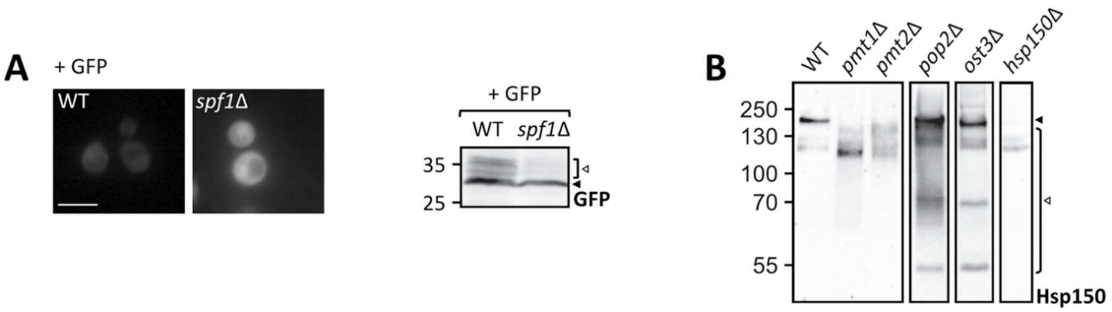

Supplementary Figure S1. Evaluation of UPOM screen hit *spf1*Δ using ER-GFP and of *pop2*Δ and *ost3*Δ using Hsp150. (**A**) Analysis of ER-GFP subcellular localization in wild type (BY4741) cells and in a screening independent *spf1*Δ strain and western blot analysis of ER-GFP O-mannosylation in total cell extracts from the same strains. Cells were transformed with ER-GFP and grown in SD supplemented with uracil for selection before being imaged under standard conditions (scale bar 5 μm) or lysed for western blot analysis. Equivalents to 0.2 OD_{600} were resolved on a 12% PAA gel and detection was performed with an anti-GFP antibody. (**B**) Western blot analysis of Hsp150 in *pop2*Δ and *ost3*Δ cells used for clustering of UPOM screen hits. Viable single deletion mutants were retrieved from the Euroscarf collection and subjected to heat shock to induce Hsp150 secretion. Proteins of the medium were resolved on 8% PAA gels and detection was performed with an anti-Hsp150 antibody. Media from wild type and *hsp150*Δ cells were included as positive controls. Hsp150 fully glycosylated and hypoglycosylated fractions are indicated with black and white arrows respectively. Results from 100 deletion mutants identified as screening hits are summarized in Supplementary Table S1.

.

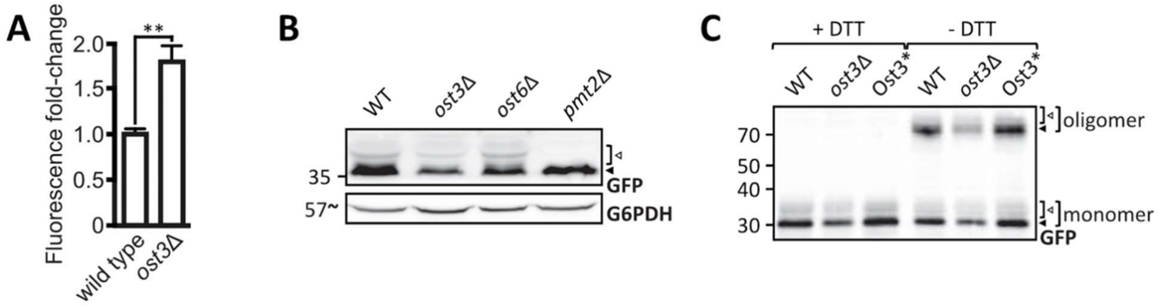

Supplementary Figure S2. Evaluation of UPOM screen hit *ost3*Δ using ER-GFP. (**A**) Flow cytometry analysis of EZY83 (wild type) and EZY82 (*ost3*Δ) cells grown to mid-log phase. Fluorescent signal resulting from analysis of 20,000 cells was normalized to wild type and results are plotted as fold-change. Error bars represent the range of values from three independent experiments ± SD. For statistical significance Tukey´s HSD test was performed. Western blot analysis of (**B**) ER-GFP O-mannosylation in total cell extracts from EZY70 (wild type), EZY77 (*ost3*Δ), EZY78 (*ost6*Δ), and *pmt2*Δ ER-GFP (*pmt2*Δ) strains and (**C**) ER-GFP oligomerization in total cell extracts from EZY83 (wild type), EZY82 (*ost3*Δ) and EZY84 (Ost3*) strains. Twenty micrograms of protein were resolved on a 12% PAA gel and detection was performed with an anti-GFP antibody. G6PDH was used as loading control. In (**C**) protein was denatured in sample buffer containing or lacking DTT. Monomeric and oligomeric ER-GFP as well as the main ER-GFP signal and higher O-mannosylated GFP-fractions are depicted by black and white arrows respectively. Asterisks report on statistical significance: ** $p \leq 0.01$.

.

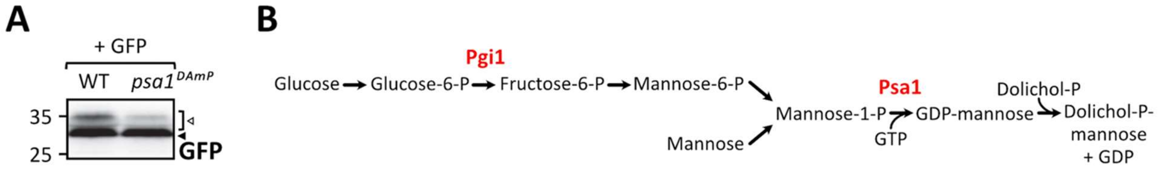

Supplementary Figure S3. Evaluation of UPOM screen hit *psa1*Δ using ER-GFP. (**A**) Western blot analysis of ER-GFP O-mannosylation in total cell extracts from wild type (BY4741) cells and the screening derived $Psa1^{DAmP}$ mutant. Equivalents to 0.2 OD_{600} were resolved on a 12% PAA gel and detection was performed with an anti-GFP antibody. Arrows on the right indicate the main GFP signal (black arrow) and signals emanating from higher O-mannosylated GFP fractions (white arrow). (**B**) Scheme of cytosolic pathways producing GDP-mannose with the UPOM screen hits Pgi1 and Psa1 highlighted in red.

.

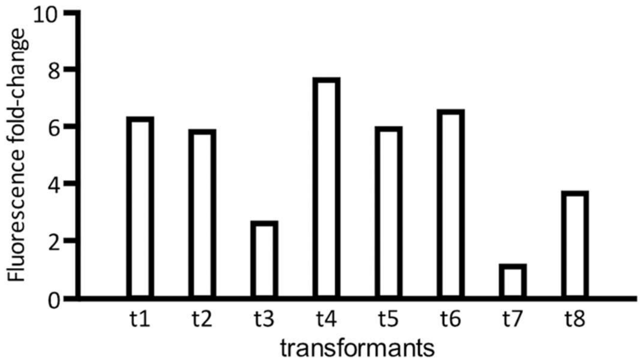

Supplementary Figure S4. Analysis of screen-independent *bfr1*Δ knockout transformants. *BFR1* was knocked out in JEY06 (wild type ER-GFP) by homologous recombination. The knockout cassette containing up- and downstream *BFR1* homologous regions and *KanMX6* was generated via PCR from pUG6. After selection, *KanMX6* insertion was verified by PCR and eight independent transformants were grown in YPD and analyzed via flow cytometry. Fluorescent signal resulting from analysis of 20,000 cells was normalized to wild type and results are plotted as fold-change.

.

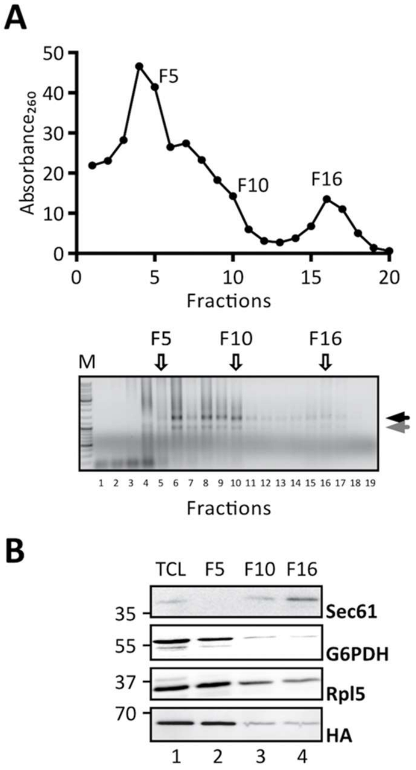

Supplementary Figure S5. Control experiment to the *PMT1* and *PMT2* transcript localization experiment depicted in Figure 6. The experiment was performed as described in Figure 6, however, in presence of EDTA. EDTA was added to total cell lysates to a final concentration of 30 mM. (**A**) EDTA treatment causes the rRNA associated $Absorbance_{260}$ to shift from ribosome associated fractions F10 (free ribosomes) and F16 (ribosomes in polysomes) to the cytoplasmic fraction F5 (upper panel) as well as disassembly of ribosomal subunits depicted by the black and grey arrow (lower panel). (**B**) In line with Bfr1 being primarily associated with ribosomes, EDTA treatment leads to redistribution of Bfr1 (HA-signal) from F10 and F16 to F5.

.

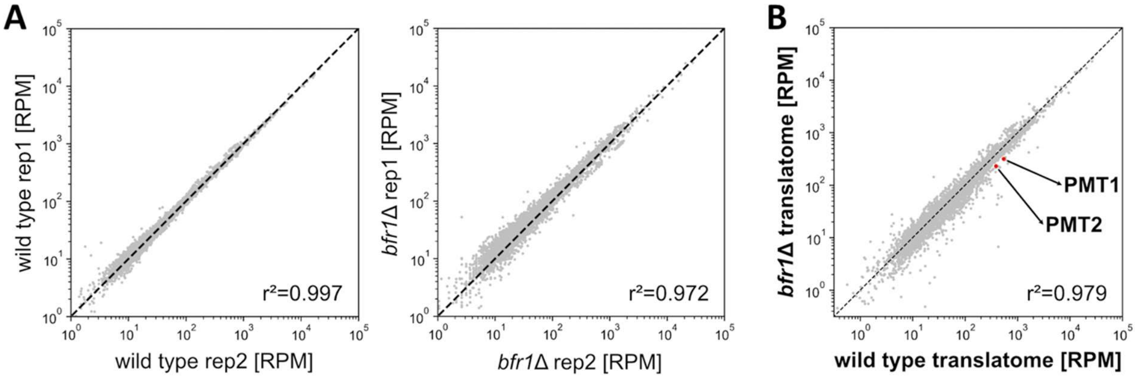

Supplementary Figure S6. Active translation of Pmt1 and Pmt2 is significantly reduced in the absence of Bfr1. (**A**) Correlation scatter plots of replicates (rep) from wild type and *bfr1*Δ cells. (**B**) Scatter plot comparing normalized ribosome densities between wild type and *bfr1*Δ cells across the *S. cerevisiae* transcriptome. Pmt1 and Pmt2 are 1.7-fold downregulated in *bfr1*Δ versus wild type cells (indicated with red dots). Data were normalized to RPMs (reads per million mapped reads).

.

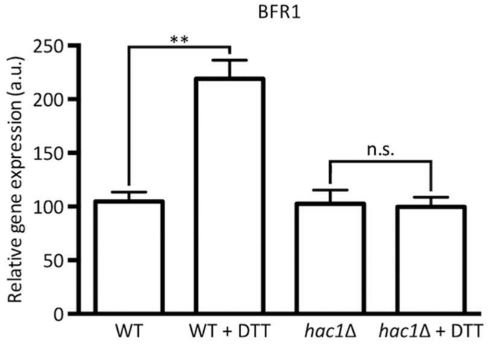

Supplementary Figure S7. *BFR1* is induced by the UPR. RT-PCR analysis of *BFR1* mRNA levels in wild type and *hac1*Δ cells in response to DTT. Wild type (BY4741) and *hac1*Δ (Euroscarf) cells were treated with 2.2 mM DTT for 60 min, total RNA was extracted, and cDNA was prepared and used as a template for RT-PCR. Results show mRNA abundance with respect to *ACT1* mRNA from three independent experiments ± SD. For statistical significance a two-tailed t-test was applied (n = 3). N.s. = not significant. Asterisks report on statistical significance: ** $p \leq 0.01$.
